# Association of Serum Uric Acid With Relative Muscle Loss: A US Population–Based Cross‐Sectional Study

**DOI:** 10.1002/jcsm.13867

**Published:** 2025-06-13

**Authors:** Fuquan Wang, Lin Wen, Xiaopeng Guo, Weiwei Wang, Yanyan Cao, Guofeng Zhou, Jun Wang, Chuansheng Zheng

**Affiliations:** ^1^ Department of Radiology Union Hospital, Tongji Medical College, Huazhong University of Science and Technology Wuhan China; ^2^ Hubei Provincial Clinical Research Center for Precision Radiology & Interventional Medicine Wuhan China; ^3^ Hubei Province Key Laboratory of Molecular Imaging Wuhan China; ^4^ Department of Nutrition and Food Hygiene, Hubei Key Laboratory of Food Nutrition and Safety, MOE Key Lab of Environment and Health School of Public Health, Tongji Medical College, Huazhong University of Science and Technology Wuhan China; ^5^ School of Food and Drug Shenzhen Polytechnic University Shenzhen China; ^6^ Department of Gastrointestinal Surgery Union Hospital, Tongji Medical College, Huazhong University of Science and Technology Wuhan China

**Keywords:** dose–response relationship, oxidative stress, relative muscle loss, sarcopenia, serum uric acid

## Abstract

**Background:**

Evidence regarding serum uric acid (SUA) and sarcopenia remains insufficient and controversial. Muscle mass is a basic and objective component of sarcopenia, and relative muscle loss has been used to define sarcopenia in some studies. We sought to investigate the association of SUA levels with relative muscle loss in the National Health and Nutrition Examination Survey (NHANES) 2011–2018.

**Methods:**

Relative muscle loss was defined by the Foundation for the National Institutes of Health (FNIH) as characterized by appendicular lean mass (ALM) adjusted by BMI (ALM/BMI) < 0.512 for women and < 0.789 for men. Multivariate logistic regression models were performed, and sample weights were accounted to reflect the nationally representative estimates. Restricted cubic spline regression was performed to visualize the dose–response relationship.

**Results:**

A total of 8967 individuals (mean age 39.4 ± 0.3 years, female 50.1%) were included, with a mean SUA of 5.3 ± 0.02 mg/dL; 762 patients with relative muscle loss (weight prevalence 7.1%) were identified, and participants in the highest quintile of SUA exhibited the highest prevalence, up to 10.5%, while participants in the lowest quintile presented the lowest prevalence (5.3%). After adjusting for sociodemographic, behavioural factors, morbidities and renal function related indicators, participants in the highest quintile of SUA levels presented an elevated risk of relative muscle loss, with OR of 1.78 (95% CI: 1.24, 2.56), as compared with the lowest quintile. This association remained stable across most subgroups, and stronger associations were observed in groups with BMI < 25 kg/m^2^ and exceeding recommended physical activity levels (*p* for interaction < 0.05). Notably, a nonlinear association between SUA and relative muscle loss was observed in the overall populations, whereas a linear association was observed in men, participants with BMI < 25 kg/m^2^, and participants with exceeding recommended physical activity levels, with the risk of relative muscle loss increasing as SUA levels increased (*p* for overall < 0.01 and *p* for nonlinear > 0.05).

**Conclusions:**

In summary, this study revealed that elevated SUA levels are a potentially independent risk factor of relative muscle loss among the US adults. Clinical screening for SUA levels may contribute to early detection and prevention of muscle loss.

## 1 Introduction

Sarcopenia is a syndrome characterized by the progressive loss of skeletal muscle mass, strength and function, occurring in ageing and other clinical conditions [1]. The occurrence of sarcopenia in adults ranged from 7% to 12%, with a steady annual increase with the growing global ageing population [2]. Increasing epidemiological evidence underscored that individuals with sarcopenia were prone to falls, disability, hospitalization and premature death, contributing to a substantial economic burden on health systems, families and individuals [3]. Sarcopenia has become a pressing public health concern worldwide. Therefore, identifying risk factors for sarcopenia is imperative to guide the implementation of early intervention strategies.

Serum uric acid (SUA), as the ultimate product of purine metabolism, has the potential to induce inflammation, oxidative stress and endothelial dysfunction [4]. Previous research indicated that higher SUA levels were closely related to increased risks of chronic kidney disease, diabetes, cardiovascular disease and metabolic disorders [5, 6]. Recent studies implied that SUA levels were also associated with sarcopenia. Several studies displayed that elevated SUA levels were associated with skeletal muscle mass loss, indicating that higher SUA levels may contribute to an increased risk of sarcopenia [7, 8]. Conversely, several studies suggested that SUA may have beneficial physiological effects as an antioxidant among participants involved from Italy [9], Brazil [10] and China [11]. In plasma, SUA neutralizes critical and hazardous pro‐oxidants such as iron‐containing radicals, peroxynitrite and hydroxyl radicals [12, 13], thereby potentially mitigating the risk of muscle mass and strength loss. These inconsistent findings prompt us to identify whether SUA can serve as a risk factor or predictor of sarcopenia among adults. Muscle mass is a basic and objective component of sarcopenia, and relative muscle loss has been used to define sarcopenia in some studies [14, 15]. To fill these knowledge gaps, we sought to investigate the association of SUA levels with relative muscle loss in the National Health and Nutrition Examination Survey (NHANES) 2011–2018 and to visualize the dose–response relationship between them.

## 2 Methods

### 2.1 Study Population

The NHANES is a large‐scale, stratified multistage and nationally representative program conducted in the United States, providing comprehensive demographic, socioeconomic, lifestyle, physical examination and laboratory data. The program was approved by the NCHS Ethics Review Board (Protocols 98–12, 2005–06, 2011–17 and 2018–01). All participants signed informed consent forms.

**FIGURE 1:**
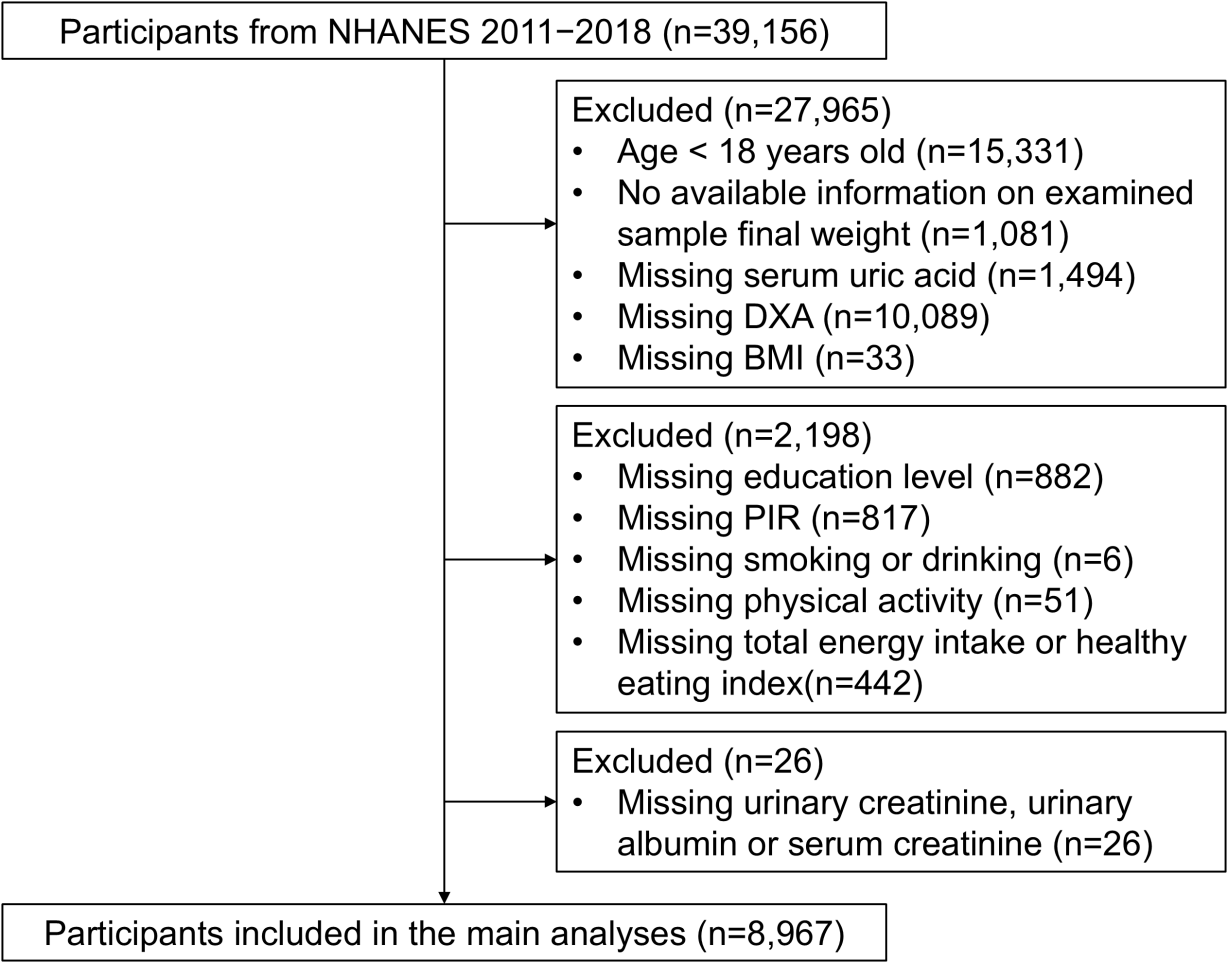
Flow chart of participants included in this study. DXA, dual‐energy X‐ray absorptiometry; BMI, body mass index; PIR, family poverty income ratio.

In this study, we utilized the data from the NHANES 2011–2018, which provided information on skeletal muscle mass assessment, measured utilizing dual‐energy X‐ray absorptiometry (DXA) scans. After excluding participants aged < 18 years, without information on SUA levels, DXA or body mass index (BMI) or without complete demographic data, 8967 participants were finally included in this study. The flow chart is presented in Figure 1.

### 2.2 Exposure Measurement

In the NHANES 2011–2016, SUA levels were measured using a Beckman UniCel DxC800 Synchron, while in the NHANES 2017–2018, a Roche Cobas 6000 analyser was used [16]. Earlier research has reported the analyses using SUA combined from multiple NHANES cycles [17].

### 2.3 Outcome Ascertainment

Appendicular lean mass (ALM) was calculated as the sum of the skeletal muscle mass of both legs and arms measured by DXA. Relative muscle loss was used as proxies for sarcopenia. According to the recommended consensus proposed by the Foundation for the National Institutes of Health (FNIH) [18], muscle loss was defined as ALM adjusted for BMI (ALM/BMI) < 0.789 for men and < 0.512 for women, consistent with previous NHANES studies [14, 15]. At present, a universally established definition for sarcopenia is notably absent, and studies have shown that different methodologies used to define sarcopenia may contribute inconsistent analysis results [19, 20]. Therefore, we further adopted two other criteria to define sarcopenia, namely: (1) sarcopenia was defined as ALM/height^2^ < 7.26 kg/m^2^ for men and < 5.45 kg/m^2^ for women based on the European Working Group on Sarcopenia in Older People (EWGSOP) and previous study utilizing NHANES [21]; (2) sarcopenia was defined as ALM < 19.75 kg for men and < 15.02 kg for women based on the alternative criteria proposed by FNIH [18]. The associations between SUA and sarcopenia (defined by different criteria) were also analysed.

Although functional assessments such as grip strength and gait speed were widely used in defining sarcopenia [22], they were not included in this study due to data availability constraints. Specifically, grip strength data were only collected in the NHANES 2011–2014 cycles, and gait speed was not assessed in NHANES during the study period (2011–2018). To ensure consistency across the entire dataset, we used relative muscle loss as proxies for sarcopenia [18]. Skeletal muscle is the most basic, objective and promising parameter among components of sarcopenia‐associated disorders. While this approach effectively evaluates muscle mass–related sarcopenia, it may not fully capture the functional aspects of the condition. Future studies incorporating functional parameters would provide a more comprehensive assessment of sarcopenia.

### 2.4 Physical Activity Assessment

The assessment of individual physical activity was based on the Global Physical Activity questionnaire (GPAQ) and GPAQ Analysis Guide of the World Health Organization (WHO) [23] and the 2018 Physical Activity Guidelines for Americans [24]. Questionnaire methods were used to investigate the frequency and duration of moderate or vigorous physical activity per week, including occupation‐related, transportation‐related and leisure‐time physical activity. Metabolic Equivalent of Task (MET) was calculated and participants were classified based on the 2018 Physical Activity Guidelines for Americans, as described in previous studies in NHANES [25]. Sufficient physical activity was defined as 150–300 min/week of moderate‐intensity, or 75–150 min/week of vigorous‐intensity physical activity, which was equivalent to 600–1200 MET‐min/week. Exceeding exercise group was defined as ≥ 300 min/week of moderate‐intensity, or ≥ 150 min/week of vigorous‐intensity physical activity (≥ 1200 MET‐min/week). Insufficient exercise group was defined as < 150 min/week of moderate‐intensity, or < 75 min/week of vigorous‐intensity physical activity (< 600 MET‐min/week). Additionally, an early guideline (2008 Physical Activity Guidelines for Americans) was further considered [26], and in this version, 150–300 min/week of moderate aerobic activity was recommended for adults. The early guideline also explicitly stated that ‘using the 2‐to‐1 rule of thumb, doing 150 minutes of vigorous‐intensity aerobic activity a week provides about the same benefits as 300 minutes of moderate intensity activity’. Details of physical activity items and suggested MET scores are shown in Table S1.

### 2.5 Covariates

Sociodemographic characteristics encompassed age, sex, race/ethnicity, education and family income. Behavioural factors and physical examination indicators included smoking status, alcohol consumption, physical activity, total energy intake (TEI), healthy eating index (HEI, calculated using HEI‐2015 [27]) and BMI. Previous studies have demonstrated the association of long‐term conditions with sarcopenia [28], so some common diseases were also included as potential confounding factors, including hypertension, dyslipidaemia, diabetes and cancer. We further considered albuminuria (characterized by a urinary albumin to creatinine ratio of > 30 mg/g) and estimated glomerular filtration rate (eGFR, calculated using the Chronic Kidney Disease Epidemiology Collaboration equation) [29]. Detailed definitions of these variables are provided in Methods S1.

### 2.6 Statistical Analyses

All statistical analyses accounted for sample weights, strata and primary sampling units to reflect the nationally representative estimates. Baseline characteristics were displayed as mean (SE) and percentages (%). Participants were grouped into quintiles 1–5 (Q1–Q5) based on the SUA levels, pursuant to the previous study in NHANES [17]. Multivariate logistic regression models were performed to explore the association between SUA levels and muscle loss, and three models were fitted. Model 1 was adjusted for age (continuous), sex and race/ethnicity. Model 2 was further adjusted for family income, education, physical activity, alcohol consumption, smoking, TEI and HEI. Model 3 was further adjusted for BMI, hypertension, dyslipidaemia, diabetes, cancer, albuminuria and eGFR. Stratified analyses were performed to explore differences among different subgroups. SAS version 9.4 (SAS Institute, USA) was utilized for these statistical analyses.

Restricted cubic spline (RCS) model was performed to visualize the dose–response associations between SUA levels and relative muscle loss in overall population and subgroups of gender, physical activity and BMI. Weights were ignored due to the absence of an available RCS model for complex, multistage sampling survey data. R 4.4.0 and R‐Studio was used for RCS models and some graphs. In this study, the RCS models of 3, 4 and 5 nodes were fitted respectively, and the optimal number of nodes was subsequently identified by the Akaike Information Criterion (AIC). AIC is a standard used to evaluate the goodness of fit and complexity of a model. The smaller the AIC value, the better the model.

## 3 Results

### 3.1 Baseline Characteristics

This study included 8967 eligible individuals (mean age 39.4 ± 0.3 years, female 50.1%) with a mean SUA level of 5.3 ± 0.02 mg/dL. The baseline characteristics according to quintiles of SUA levels are exhibited in Table 1. Notably, a significant difference in SUA levels was observed in gender subgroups, with males having higher SUA levels. Following comparing differences in behaviour‐related factors, long‐term conditions and renal function related indicators, participants with higher SUA levels were characterized as smokers, drinkers, with exceeding recommended physical activity levels, high TEI, low HEI, obese and having hypertensive, dyslipidaemia or proteinuria.

**TABLE 1:** Baseline characteristics of participants.

	SUA levels, mg/dL (*n* = 8967)						
	Overall	Q1	Q2	Q3	Q4	Q5	
Characteristic	5.32 (0.02)	< 4.1	4.1–4.8	4.9–5.5	5.6–6.4	> 6.4	*p*
**Participants, *n***	8967	1842	1697	1789	1829	1810	
**Age, years**	39.40 (0.27)	39.33 (0.37)	39.48 (0.41)	39.26 (0.42)	39.73 (0.50)	39.19 (0.39)	< 0.001
**Women, *n* (%)**	4582 (50.14)	1597 (88.33)	1190 (70.82)	904 (48.89)	606 (30.35)	285 (14.12)	< 0.001
**Race or ethnicity, *n* (%)**							
Non‐Hispanic White	3305 (63.23)	665 (62.02)	607 (63.08)	668 (63.29)	687 (64.00)	678 (63.72)	0.302
Non‐Hispanic Black	1827 (10.62)	367 (10.98)	352 (10.77)	374 (10.79)	348 (9.52)	386 (11.11)	
Mexican American or Hispanic	2205 (16.96)	511 (18.73)	423 (16.14)	436 (16.72)	465 (17.62)	370 (15.52)	
Other	1630 (9.19)	299 (8.27)	315 (10.01)	311 (9.20)	329 (8.86)	376 (9.65)	
**Education level, *n* (%)**							
Less than high school	1497 (11.85)	316 (11.51)	287 (11.92)	285 (11.56)	323 (12.59)	286 (11.65)	0.183
High school	1953 (21.64)	367 (19.86)	364 (19.49)	397 (22.49)	406 (22.62)	419 (23.58)	
College or higher	5517 (66.51)	1159 (68.63)	1046 (68.59)	1107 (65.95)	1100 (64.79)	1105 (64.77)	
**PIR, *n* (%)**							
< 1.31	2900 (23.09)	637 (25.71)	571 (23.38)	554 (21.30)	590 (23.24)	548 (21.83)	0.314
1.31–3.50	3236 (34.81)	668 (33.87)	582 (33.57)	659 (35.47)	675 (36.08)	652 (34.96)	
> 3.50	2831 (42.10)	537 (40.42)	544 (43.05)	576 (43.23)	564 (40.68)	610 (43.21)	
**Smoking status, *n* (%)**							
Nonsmoker	5434 (59.08)	1194 (62.21)	1055 (58.27)	1111 (61.35)	1064 (57.64)	1010 (55.94)	< 0.001
Former smoker	1528 (19.71)	228 (14.06)	272 (19.94)	293 (19.66)	329 (21.12)	406 (23.74)	
Current smoker	2005 (21.21)	420 (23.73)	370 (21.79)	385 (18.99)	436 (21.24)	394 (20.32)	
**Alcohol intake, *n* (%)**							
None	6707 (70.57)	1495 (76.03)	1322 (73.21)	1343 (72.98)	1326 (68.31)	1221 (62.51)	< 0.001
Moderate	628 (7.49)	82 (5.23)	112 (7.42)	105 (5.71)	158 (9.12)	171 (9.88)	
Heavy	1632 (21.94)	265 (18.74)	263 (19.37)	341 (21.31)	345 (22.57)	418 (27.61)	
**Physical activity, *n* (%)**							
Insufficient	2768 (28.10)	628 (30.59)	561 (29.24)	538 (28.03)	508 (25.29)	533 (27.44)	< 0.001
Sufficient	927 (10.12)	221 (12.95)	162 (9.80)	180 (8.82)	187 (9.38)	177 (9.69)	
Exceeding	5272 (61.80)	993 (56.46)	974 (60.95)	1071 (63.15)	1134 (65.33)	1100 (62.86)	
**TEI, kcal/day**	2263.86 (12.56)	2066.39 (30.05)	2145.25 (28.47)	2304.61 (26.43)	2360.11 (26.50)	2433.02 (44.10)	< 0.001
**HEI**	52.50 (0.29)	53.30 (0.58)	54.11 (0.55)	52.90 (0.42)	52.45 (0.37)	50.87 (0.36)	< 0.001
**BMI, kg/m** ^**2**^							
< 25.0	2809 (31.12)	866 (49.79)	637 (38.89)	567 (30.65)	450 (23.50)	289 (13.44)	< 0.001
25.0–29.9	2815 (32.44)	530 (29.44)	522 (31.25)	561 (32.38)	593 (33.03)	609 (36.03)	
≥ 30.0	3343 (36.44)	446 (20.77)	538 (29.86)	661 (36.97)	786 (43.47)	912 (50.52)	
**Hypertension, *n* (%)**	2521 (26.69)	373 (20.92)	389 (23.31)	539 (30.69)	729 (38.79)	883 (47.92)	< 0.001
**Dyslipidaemia, *n* (%)**	2913 (32.46)	469 9 (27.41)	473 (29.34)	567 (33.31)	615 (32.19)	737 (41.11)	< 0.001
**Diabetes, *n* (%)**	958 (8.28)	173 (7.31)	154 (6.97)	197 (8.74)	207 (8.51)	227 (9.81)	0.070
**Cancer, *n* (%)**	342 (4.85)	79 (5.88)	59 (4.46)	76 (5.07)	63 (4.28)	65 (4.57)	0.646
**eGFR, mL/min/1.73 m** ^**2**^	93.83 (0.48)	99.95 (0.81)	95.82 (0.71)	94.33 (0.78)	91.00 (0.66)	88.24 (0.72)	< 0.001
**Albuminuria, *n* (%)**	720 (6.59)	155 (7.08)	107 (5.09)	123 (5.79)	133 (5.94)	202 (9.04)	< 0.001

*Note:* Values are weighted mean (SE) for continuous variables or numbers (weighted percentages %) for categorical variables. PIR, family poverty income ratio; TEI, total energy intake; HEI, healthy eating index; BMI, body mass index; eGFR, estimated glomerular filtration rate.

### 3.2 Association of SUA Levels With Muscle Loss

A total of 762 patients with relative muscle loss were observed, with a weighted prevalence rate of 7.1%. Participants with SUA levels in the highest quintile exhibited the highest incidence rate of relative muscle loss, up to 10.5%, while participants in the lowest quintile presented the lowest incidence rate (5.3%). After simply adjusting for age, sex and race/ethnicity, participants in the highest quintile of SUA levels exhibited an elevated risk of relative muscle loss, with OR of 2.70 (95% CI: 1.87, 3.90), as compared with the lowest quintile. Following comprehensive adjusting for sociodemographic, behavioural factors, long‐term morbidities and renal function related indicators, the association of SUA levels with relative muscle loss risk remained significant, with OR of 1.78 (95% CI: 1.24, 2.56). Notably, as SUA levels rose, the risk of relative muscle loss exhibited an increasing trend (*p* for trend = 0.004). For a one‐unit increment in SUA levels, the OR of relative muscle loss was 1.19 (95% CI: 1.08, 1.31). Detailed results of all three fitted models are shown in Table 2 and Figure S1.

**TABLE 2:** Associations of SUA levels with relative muscle loss in the NHANES 2011–2018.

	SUA levels, mg/dL						
	Q1	Q2	Q3	Q4	Q5	Per 1 mg/dL increment	*p* for trend
	< 4.1	4.1–4.8	4.9–5.5	5.6–6.4	> 6.4		
No. case/total	135/1842	117/1697	152/1789	156/1829	202/1810	—	
Prevalence (%)	5.26	5.58	6.65	7.36	10.53	—	
Model 1	1 (Ref)	1.121 (0.758, 1.658)	1.435 (0.994, 2.071)	1.623 (1.152, 2.286)**	2.698 (1.868, 3.896)***	1.291 (1.181, 1.410)***	< 0.001
Model 2	1 (Ref)	1.127 (0.744, 1.708)	1.382 (0.962, 1.985)	1.511 (1.070, 2.135)*	2.490 (1.739, 3.564)***	1.259 (1.155, 1.373)***	< 0.001
Model 3	1 (Ref)	0.995 (0.632, 1.568)	1.097 (0.745, 1.617)	1.136 (0.796, 1.621)	1.778 (1.235, 2.558)**	1.190 (1.079, 1.312)***	0.004

*Note:* Values are n, weighted percentages, or weighted OR (95% CI). Ref, reference. *p < 0.05, **p < 0.01, ***p < 0.001.

Model 1 was adjusted for age (continuous), sex and race or ethnicity.

Model 2 was further adjusted for education level, family income level, smoking status, alcohol intake, physical activity, total energy intake (in quartiles) and HEI (in quartiles).

Model 3 was adjusted for model 2 plus BMI (< 25, 25–29.9 or ≥ 30 kg/m^2^), hypertension, dyslipidaemia, diabetes, cancer, albuminuria and eGFR.

Table S2 shows the associations of SUA levels with sarcopenia defined by absolute muscle mass and height‐adjusted muscle mass. After adjusting for BMI (as a categorical variable, divided into < 25, 25–29.9 or ≥ 30 kg/m^2^) and other confounding factors, no significant association was observed between SUA levels and low absolute muscle mass risk. Following fully adjusting for BMI (as a continuous variable) and other confounding factors, participants in the highest quintile of SUA levels exhibited an elevated risk of low absolute muscle mass, with OR of 2.26 (95% CI: 1.33, 3.82), as compared with the lowest quintile. Similarly, participants in the highest quintile of SUA levels (vs. quintile 1) were significantly associated with low height‐adjusted muscle mass risk, showing an OR of 2.79 (95% CI: 1.84, 4.24) after fully adjusting for BMI (as a continuous variable) and other confounding factors.

### 3.3 Subgroup Analyses

In stratified analysis, individuals with elevated SUA levels consistently demonstrated a significant association with increased risks of relative muscle loss across most subgroups. In the male group, participants in the highest quintile of SUA levels (vs. quintile 1) were significantly associated with relative muscle loss risk, showing an OR of 2.25 (95% CI: 1.29, 3.90), while the association was not significant in women with OR of 1.40 (95% CI: 0.83, 2.34), and the test for multiplicative interactions is not statistically significant (*p* for interaction = 0.478). Subsequent analyses of other subgroups revealed significant interactions of SUA levels with physical activity and BMI for relative muscle loss (*p* for interaction < 0.05, 0.01, respectively), and did not show the interaction among other variables (*p* for interaction > 0.05). The ORs of participants in the highest quintile SUA levels (vs. quintile 1) and relative muscle loss risk were 3.00 (95% CI: 1.67, 5.38, highest among groups classified by physical activity) in participants with exceeding recommended physical activity levels, and 10.13 (95% CI: 2.83, 36.23, highest among groups classified by BMI) in participants with a BMI < 25 kg/m^2^. Detailed results of the subgroup analyses are presented in Table S3 and Figure S2.

### 3.4 Dose–Response Relationship Between SUA Levels and Relative Muscle Loss

**FIGURE 2:**
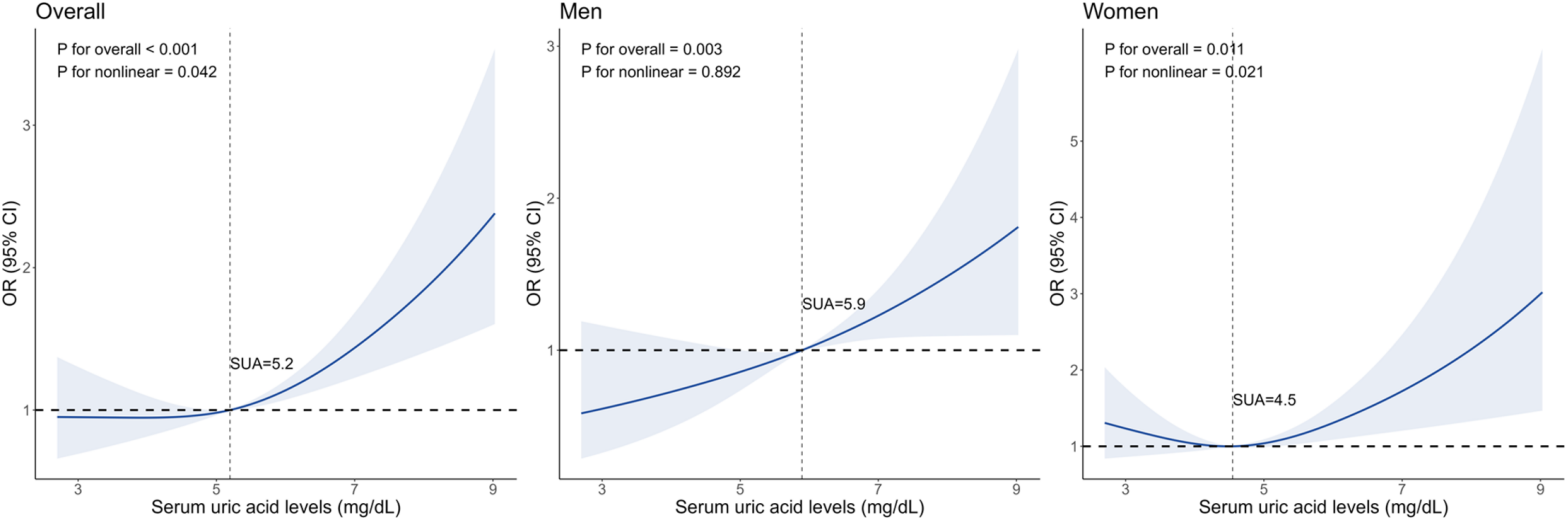
Restricted cubic spline plots of the association between SUA levels and relative muscle loss. Models were adjusted for age (continuous), sex (except in the gender subgroup analyses), race/ethnicity, education, family income, smoking status, alcohol intake, physical activity, total energy intake (in quartiles), healthy eating index (in quartiles), BMI (< 25, 25–29.9 or ≥ 30 kg/m^2^), hypertension, dyslipidaemia, diabetes, cancer, albuminuria and eGFR.

**FIGURE 3:**
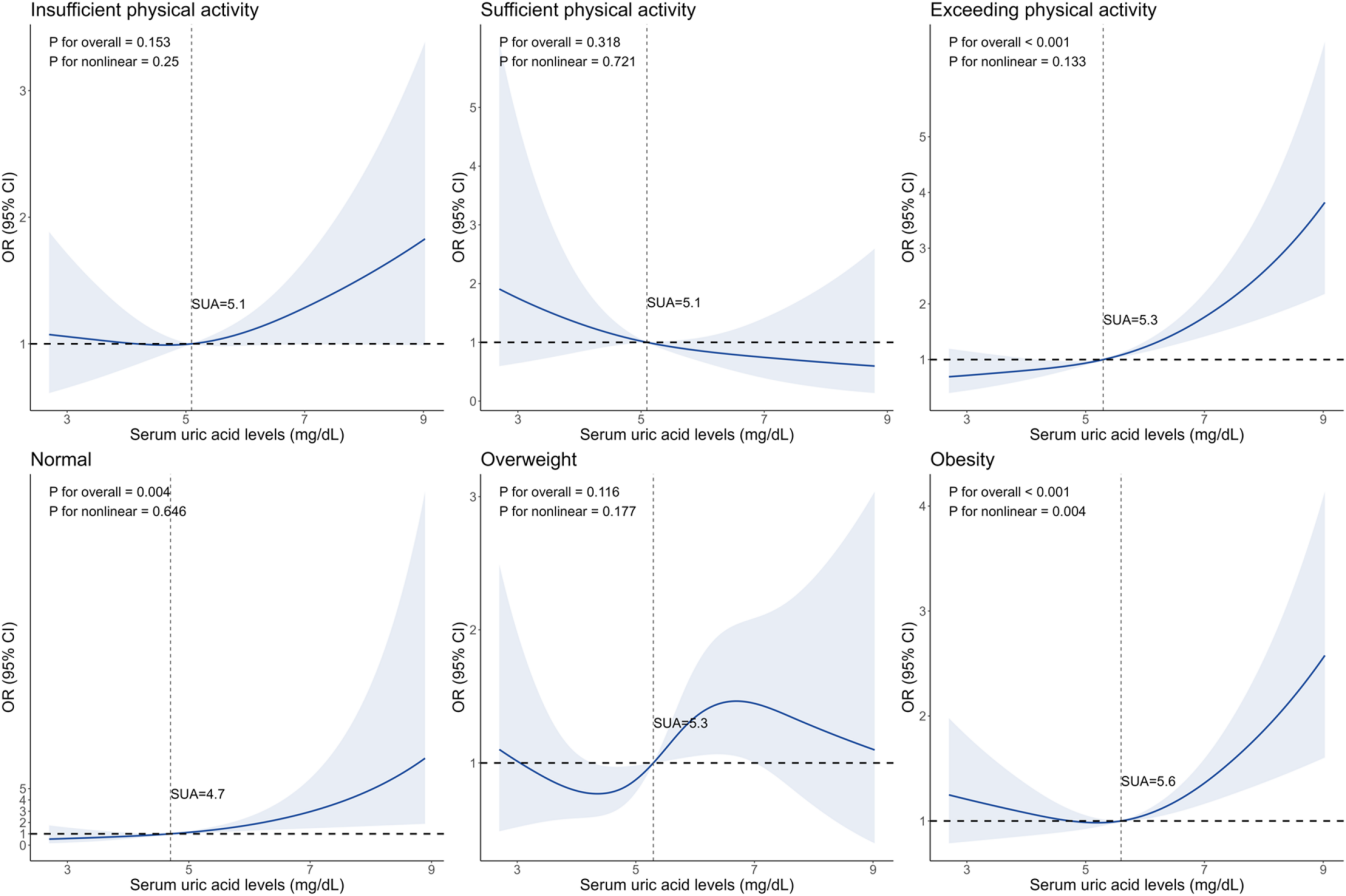
Restricted cubic spline plots of the association between SUA levels and relative muscle loss in subgroups classified by physical activity and BMI. Models were adjusted for age (continuous), sex, race/ethnicity, education, family income, smoking status, alcohol intake, physical activity (except in the physical activity subgroup analyses), total energy intake (in quartiles), healthy eating index (in quartiles), BMI (< 25, 25–29.9 or ≥ 30 kg/m^2^, except in the BMI subgroup analyses), hypertension, dyslipidaemia, diabetes, cancer, albuminuria and eGFR.

RCS models were performed to visualize the dose–response associations between SUA levels and relative muscle loss in overall population and subgroups classified by gender, physical activity and BMI. A nonlinear dose–response curve was observed in 8967 individuals, with the risk of relative muscle loss increasing as SUA levels increased (*p* for nonlinear < 0.05). In stratified analyses of gender, a similar nonlinear dose–response association was observed in women (*p* for nonlinear < 0.05), whereas a linear dose–response association was observed in men (*p* for overall < 0.01 and *p* for nonlinear > 0.05) (Figure 2). In stratified analyses of groups classified by physical activity levels and BMI, linear dose–response associations were observed in participants with exceeding recommended physical activity levels, and participants with a BMI < 25 kg/m^2^, both with an increasing risk of relative muscle loss as SUA levels increased (*p* for overall < 0.01 and *p* for nonlinear > 0.05) (Figure 3).

**FIGURE 4:**
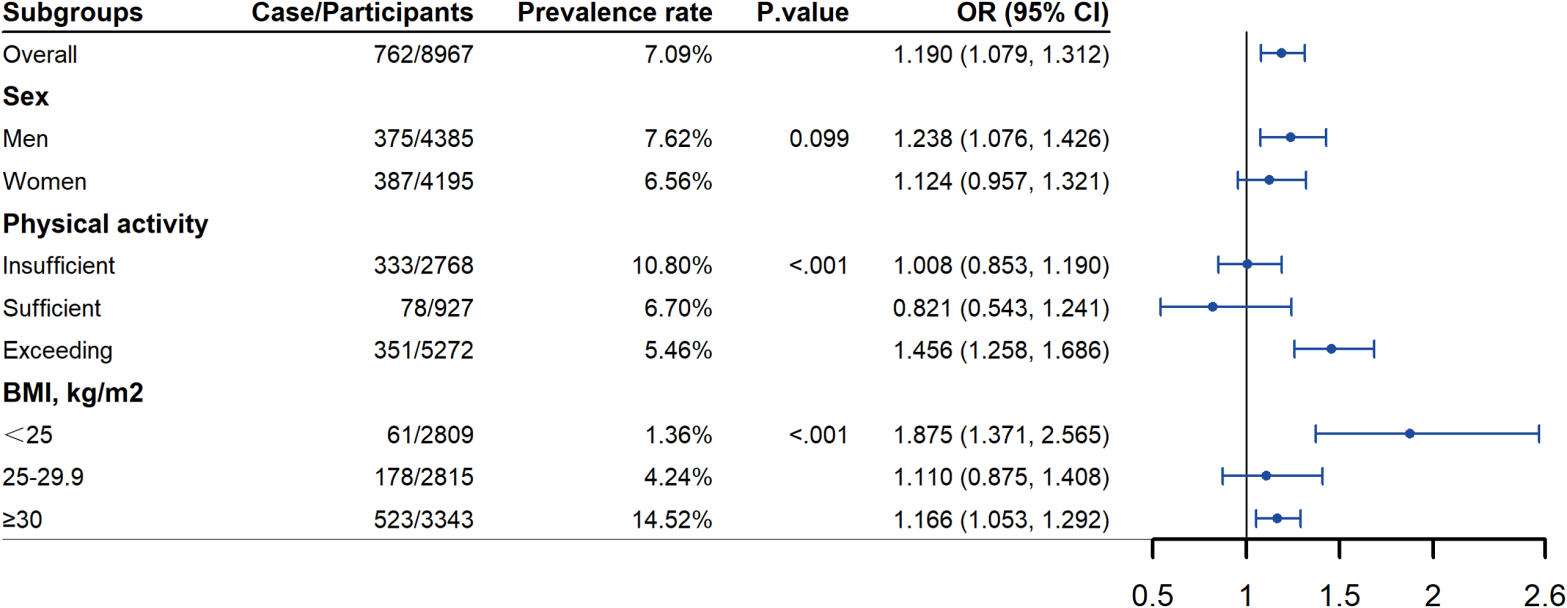
Associations of SUA levels with relative muscle loss based on subgroup analyses of gender, physical activity and BMI. All statistical analyses accounted for sample weights, strata and primary sampling units to reflect the nationally representative estimates. *p* values show the results of the *χ* ^2^ tests, which are used to examine differences in the prevalence of relative muscle loss among subgroups. ORs (odds ratios) show the associations of serum uric acid (continuous) with relative muscle loss in subgroups. Models were adjusted for age (continuous), sex (except in the gender subgroup analyses), race/ethnicity, education, family income, smoking status, alcohol intake, physical activity (except in the physical activity subgroup analyses), total energy intake (in quartiles), healthy eating index (in quartiles), BMI (< 25, 25–29.9 or ≥ 30 kg/m^2^, except in the BMI subgroup analyses), hypertension, dyslipidaemia, diabetes, cancer, albuminuria and eGFR.

Generalized linear models were used to calculate the detailed elevated risk of relative muscle loss as SUA levels increased in subgroups divided by gender, physical activity and BMI. After fully adjusting for confounder factors, and accounting for sample weights, strata and primary sampling units, for a one‐unit increment in SUA levels, the ORs of relative muscle loss were 1.24 (95% CI: 1.08, 1.43) in men, 1.46 (95% CI: 1.26, 1.69) in participants with exceeding recommended physical activity levels, and 1.88 (95% CI: 1.37, 2.57) in participants with a BMI < 25 kg/m^2^. Details are presented in Figure 4.

## 4 Discussion

Leveraging data from representative populations in the United States, we identified a significant positive association between high SUA levels and an increased risk of relative muscle loss. This association remained stable across most subgroups, and stronger associations were observed in groups with BMI < 25 kg/m^2^ and exceeding recommended physical activity levels. A nonlinear association between SUA and relative muscle loss in the overall population was observed, whereas a linear association was observed in men, participants with BMI < 25 kg/m^2^, and participants with exceeding recommended physical activity levels. These results highlight the clinical value of high SUA levels as an independent risk factor and potential blood biomarker for muscle loss.

As a common clinical indicator of purine metabolism, the relationship between SUA and sarcopenia has been widely discussed in recent years. A study conducted in Japan observed a significantly lower muscle strength in the hyperuricaemia group compared with the nonhyperuricaemia group among 586 men [8]. Beavers et al.'s study demonstrated that participants in the highest SUA concentrations group had a 2.0‐fold risk of sarcopenia compared with the lowest SUA group [7], which was similar to the 1.8‐fold risk found in our study. The evidence suggests that high SUA may serve as a potential risk factor for sarcopenia. However, Can et al.'s study with 72 patients recruited from the geriatric outpatient clinic observed significantly lower SUA levels in patients with sarcopenia [30]. A study conducted in West China found a notable negative correlation between high SUA levels and sarcopenia in both genders, suggesting that high SUA levels may serve as an independent protective factor for muscle mass and strength [31]. Nahas et al.'s study found no significant association of SUA with appendicular muscle mass index [32]. The current evidence in this field is still insufficient and controversial, and differences in sarcopenia definition criteria, adjustment for confounders, and ethnic and regions may potentially contribute to the inconsistent conclusions. At present, a universally established definition for sarcopenia is notably absent, which may be due to the diversity of muscle mass measurements. Earlier studies highlighted that individual differences were an important factor influencing muscle mass, especially in gender, height and weight [18]. The commonly used definition criteria for sarcopenia mainly include the consensus proposed by FNIH, EWGSOP and Asian Working Group for Sarcopenia (AWGS) respectively, which primarily used BMI‐adjusted muscle mass (ALM/BMI) and height‐adjusted muscle mass (ALM/height^2^). Peng et al.'s and Takegami et al.'s studies have shown that different methodologies used to define sarcopenia may contribute to inconsistent results [19, 20]. These prompted us to re‐examine which definition was more appropriate and whether the definition chosen accurate for a specific population and study.

In this study, the associations of SUA with absolute and relative muscle mass loss were analysed and compared, and BMI was identified as a crucial factor contributing to the inconsistent results. Adjusting for BMI in a coarse category may be insufficient in studies of sarcopenia defined using absolute muscle mass or ALM/height^2^. Furthermore, ALM/BMI combined height and weight information, and the results appeared to be more robust. ALM/BMI was recommended as a sarcopenia‐related indicator by the FNIH in 2014 [18]. Subsequently, Moon Joon‐Ho et al. conducted a community‐based prospective cohort study and found that ALM/BMI provided better prognostic values than ASM/height^2^ in long‐term mortality risk [33]. Additionally, AWGS 2019 indicated that ALM/BMI may be superior to unadjusted muscle mass (or ALM/height^2^) in predicting functional outcomes or adverse clinical outcomes, encouraging the use of ALM/BMI in future studies to determine the best way to measure muscle mass [34].

Notably, renal function related indicators are important confounding factors, which should be considered but were ignored in previous study [31]. Evidence suggested that renal function was closely related to SUA and sarcopenia [35]. Renal dysfunction may be accompanied by sarcopenia, and patients with chronic kidney disease may have higher SUA levels because of limited SUA excretion [36]. Adjusting indicators related to renal function may contribute to identifying whether SUA levels could be used as an independent risk factor for sarcopenia. Leveraging data from a large and nationally representative population in the United States and muscle mass as assessed by DXA, we observed SUA as a potential independent risk factor for relative muscle loss with adjustments of various confounding factors, including renal function related indicators.

Considering that the average SUA levels of diverse populations is different (e.g., the SUA level of Japanese who eat more seafood was higher than that of participants in western China), it may be limited in generalizability to simply divide the study population into high/low groups or quartiles according to SUA levels. However, previous studies have mostly categorized SUA levels as either high or low, or into quartiles [8, 11, 32], so the dose–response relationship between SUA levels and sarcopenia remains unclear. To address this issue, we further performed RCS regression analyses and found a nonlinear association between them, with the risk of muscle loss increasing as SUA levels increased. This provides scientific evidence to clarify the relationship between SUA and sarcopenia.

Several underlying mechanisms might explain the association between high SUA levels and sarcopenia risk. First, UA can trigger inflammation by fostering the generation of reactive oxygen species (ROS) in muscle tissue. While functioning as a systemic circulation antioxidant via pro‐oxidant neutralization, UA exerts pro‐oxidant intracellular effects, triggering ROS production through three distinct pathways: (1) stimulation of membrane‐bound NADPH oxidase‐mediated superoxide (O_2_^−^•) synthesis through catalytic NADPH conversion [37], (2) upregulation of xanthine oxidase activity in the metabolic process of UA [38] and (3) mitochondrial electron transport chain impairment that induces electron leakage and consequent superoxide overproduction [12]. This process leads to damage and functional abnormalities in skeletal muscle cells, which are central to the development of sarcopenia. Second, elevated SUA levels may cause endothelial dysfunction, resulting in vascular disease that hampers blood flow and nutrient supply, thereby intensifying skeletal muscle mass loss [39]. Third, high SUA levels can detrimentally impact kidney function, disrupting the body's metabolic cycle and its ability to maintain fluid balance, which further contributes to the decline in muscle strength [40]. Finally, elevated SUA is also linked to insulin resistance, which can lead to the breakdown of muscle proteins and a decrease in physical capacity—key factors in the onset of sarcopenia [41]. Together, these pathways highlight the complex role of SUA in facilitating skeletal muscle mass loss and sarcopenia within the population.

Interestingly, in subgroup analyses, we found that higher SUA levels were more strongly associated with an increased risk of relative muscle loss among individuals with exceeding recommended exercise levels. Previous studies showed that strengthening nutrition and exercise could contribute to preventing and improving sarcopenia [1, 42]. This insight was supported by the data from our study, as we observed that the prevalence of relative muscle loss in the exceeding exercise group was lowest among the three groups divided by physical exercise levels. However, some vigorous exercise may cause muscle strain, and increase oxidative injury, and accelerate purine nucleotide degradation to form uric acid [43]. These may contribute to a strong association between SUA and sarcopenia in excess exercise groups. Therefore, while physical exercise is recommended, monitoring SUA levels is needed for individuals who regularly engage in high‐intensity exercise to mitigate skeletal muscle strain and sarcopenia risk. Additionally, among groups classified by BMI, we observed the strongest association between SUA levels and relative muscle loss in participants with a BMI < 25 kg/m^2^. In fact, some participants with low BMI may be caused by serious medical conditions, which may be accompanied by muscle mass loss, cachexia, chronic inflammation and elevated SUA levels [44, 45]. These may partly explain the strong association in low BMI group. It is worth noting that the low incidence of outcomes in the low BMI group may limit the statistical accuracy of the association.

Our study possesses two noteworthy strengths. First, we leveraged data from representative populations in the United States and considered sample weights in statistical analyses, thereby furnishing scientific and persuasive evidence in this regard. Second, utilizing the high‐quality data, we were able to consider numerous confounders, particularly renal function related indicators, often neglected in previous studies. However, several limitations warrant consideration. First, given the cross‐sectional design of our research, direct causality inference should be cautious. Second, the statistical power is restricted in detecting weak or moderate differences in the subgroup analyses, necessitating cautious interpretation of the results, especially for subgroup results from RCS models, as larger sample sizes are required due to the complexity of the models. Third, relative muscle loss was used as proxies for sarcopenia, which may not fully capture the functional aspects of the condition. Future studies incorporating functional parameters would provide a more comprehensive assessment of sarcopenia. Finally, although we have considered TEI and HEI, purine intake was not adjusted due to the difficulty in obtaining accurate and detailed food purine intake. SUA may play a mediating role between nutrient intake and the development of sarcopenia, which is a potentially promising direction for future research.

In summary, this study revealed that an elevated SUA level is a potential independent risk factor associated with relative muscle loss among the US adults. Clinical screening for SUA levels may contribute to early detection and prevention of muscle loss. Future prospective studies are essential to explore whether reducing urate‐related dietary intake and managing SUA levels can decrease the prevalence of muscle loss and sarcopenia.

## Conflicts of Interest

The authors declare no conflicts of interest.

## Supporting information


**Table S1.** Physical activity assessment in the NHANES 2011–2018.
**Table S2.** Associations of SUA levels with absolute muscle mass and height‐adjusted muscle mass in the NHANES 2011–2018. Values are weighted OR (95% CI). Ref, reference. * < 0.05, ** < 0.01, *** < 0.001. Low absolute muscle mass was defined as ALM < 19.75 kg for men and < 15.02 kg for women based on the Foundation for the National Institutes of Health (FNIH). Low height‐adjusted muscle mass was defined as ALM/height‍^2^ < 7.26 kg/m^2^ for men and < 5.45 kg/m‍^2^ for women based on the European Working Group on Sarcopenia in Older People (EWGSOP), and previous study utilizing NHANES (Karanth, Shama D et al., 2021). All statistical analyses accounted for sample weights, strata, and primary sampling units to reflect the nationally representative estimates. Model 1 was adjusted for age (continuous), sex, race or ethnicity, education level, family income level, smoking status, alcohol intake, physical activity, total energy intake (in quartiles), healthy eating index (in quartiles), BMI (< 25, 25–29.9, or ≥ 30 kg/m^2^) hypertension, dyslipidaemia, diabetes, cancer, albuminuria, and eGFR. Model 2 was adjusted for age (continuous), sex, race or ethnicity, education level, family income level, smoking status, alcohol intake, physical activity, total energy intake (in quartiles), healthy eating index (in quartiles), BMI (continuous), hypertension, dyslipidaemia, diabetes, cancer, albuminuria, and eGFR.
**Table S3.** Subgroup analysis of the association between SUA levels and relative muscle loss in the NHANES 2011–2018. Values are weighted odds ratios (95% confidence intervals). All statistical analyses accounted for sample weights, strata, and primary sampling units to reflect the nationally representative estimates. Models were adjusted for age (continuous), sex, race or ethnicity, education level, family income level, smoking status, alcohol intake, physical activity, total energy intake (in quartiles), HEI‐2015 (in quartiles), BMI (< 25, 25–29.9, or ≥ 30 kg/m^2^), hypertension, dyslipidaemia, diabetes, cancer, albuminuria, and eGFR. The subgroup variables themselves were not included as covariables in their respective models. * < 0.05, ** < 0.01.
**Figure S1.** Forest plot of association between SUA levels and relative muscle loss in the NHANES 2011–2018. Quintile 1 were used as a reference. Model 1 was adjusted for age (continuous), sex, and race or ethnicity. Model 2 was further adjusted for education level, family income level, smoking status, alcohol intake, physical activity, total energy intake (in quartiles), HEI‐2015 (in quartiles). Model 3 was adjusted for model 2 plus BMI (< 25, 25–29.9, or ≥ 30 kg/m^2^), hypertension, dyslipidaemia, diabetes, cancer, albuminuria, and eGFR.
**Figure S2.** Forest plot of subgroup analysis results on the association between SUA levels and relative muscle loss in the NHANES 2011–2018. Quintile 1 were used as a reference.
