# Supplementary material for: Association of Serum Uric Acid With Relative Muscle Loss: A US Population–Based Cross‐Sectional Study
Source: J Cachexia Sarcopenia Muscle. 2025 Jun 13;16(3):e13867. doi: 10.1002/jcsm.13867 (PMC12163516; doi:10.1002/jcsm.13867)
Supplement: Supplementary file 1 — Table S1. Physical activity assessment in the NHANES 2011–2018. Table S2. Associations of SUA levels with absolute muscle mass and height‐adjusted muscle mass in the NHANES 2011–2018. Values are weighted OR (95% CI). Ref, reference. * < 0.05, ** < 0.01, *** < 0.001. Low absolute muscle mass was defined as ALM < 19.75 kg for men and < 15.02 kg for women based on the Foundation for the National Institutes of Health (FNIH). Low height‐adjusted muscle mass was defined as ALM/height‍2 < 7.26 kg/m2 for men and < 5.45 kg/m‍2 for women based on the European Working Group on Sarcopenia in Older People (EWGSOP), and previous study utilizing NHANES (Karanth, Shama D et al., 2021). All statistical analyses accounted for sample weights, strata, and primary sampling units to reflect the nationally representative estimates. Model 1 was adjusted for age (continuous), sex, race or ethnicity, education level, family income level, smoking status, alcohol intake, physical activity, total energy intake (in quartiles), healthy eating index (in quartiles), BMI (< 25, 25–29.9, or ≥ 30 kg/m2) hypertension, dyslipidaemia, diabetes, cancer, albuminuria, and eGFR. Model 2 was adjusted for age (continuous), sex, race or ethnicity, education level, family income level, smoking status, alcohol intake, physical activity, total energy intake (in quartiles), healthy eating index (in quartiles), BMI (continuous), hypertension, dyslipidaemia, diabetes, cancer, albuminuria, and eGFR. Table S3. Subgroup analysis of the association between SUA levels and relative muscle loss in the NHANES 2011–2018. Values are weighted odds ratios (95% confidence intervals). All statistical analyses accounted for sample weights, strata, and primary sampling units to reflect the nationally representative estimates. Models were adjusted for age (continuous), sex, race or ethnicity, education level, family income level, smoking status, alcohol intake, physical activity, total energy intake (in quartiles), HEI‐2015 (in quart [file JCSM-16-e13867-s001.docx]

**Supplementary Methods**

**Covariates**

Family income-to-poverty ratios were categorized as ≤ 1.30, 1.31–3.50, and > 3.50, and a higher ratio represents a better family economic status^1^. Smoking status was classified as current smokers (individuals with > 100 cigarettes lifetime consumption), former smokers (individuals with > 100 cigarettes consumption who quit), and nonsmokers (individuals with < 100 cigarettes consumption). Alcohol consumption was classified as nondrinkers, low-to-moderate drinkers (< 2 drinks/day in men, < 1 drink/day in women), or heavy drinkers (≥ 2 drinks/day in men, ≥ 1 drinks/day in women).

Hypertension was defined as taking blood pressure medication or a diastolic blood pressure ≥ 90 mmHg or a systolic blood pressure ≥ 140 mmHg^2^. Hyperlipidemia was defined as a self-reported doctor diagnosis, triglycerides (TG) ≥ 150 mg/dL, or high density lipoprotein cholesterol (HDL-C) < 40mg/d^3^. Diabetes was defined as a self-reported doctor diagnosis, use of insulin or oral hypoglycemic medication, fasting blood glucose ≥ 7.0 mmol/L, postprandial plasma glucose ≥ 11.1 mmol/L, or glycated hemoglobin A1c (HbA1c) levels ≥ 6.5%^4^. Cancer was identified by self-reported doctor diagnosis.

**Reference**

1. Johnson, C. L. et al. National health and nutrition examination survey: analytic guidelines, 1999-2010. Vital Health Stat. 2. 1–24 (2013).

2. Whelton, P. K. et al. 2017 ACC/AHA/AAPA/ABC/ACPM/AGS/APhA/ASH/ASPC/NMA/PCNA Guideline for the Prevention, Detection, Evaluation, and Management of High Blood Pressure in Adults: A Report of the American College of Cardiology/American Heart Association Task Force on Clinical Practice Guidelines. Circulation 138, e484–e594 (2018).

3. National Cholesterol Education Program (NCEP) Expert Panel on Detection, Evaluation, and Treatment of High Blood Cholesterol in Adults (Adult Treatment Panel III). Third Report of the National Cholesterol Education Program (NCEP) Expert Panel on Detection, Evaluation, and Treatment of High Blood Cholesterol in Adults (Adult Treatment Panel III) final report. Circulation 106, 3143–3421 (2002).

4. Zhang, N. et al. Type 2 diabetes mellitus unawareness, prevalence, trends and risk factors: National Health and Nutrition Examination Survey (NHANES) 1999-2010. J. Int. Med. Res. 45, 594–609 (2017).

**Table S1.** **Physical activity assessment in the NHANES 2011–2018.**

|  | Items | Questionnaire | Suggested MET scores | Codes |
| --- | --- | --- | --- | --- |
| 1 | Vigorous work activity | 1). Does your work involve vigorous-intensity activity that causes large increases in breathing or heart rate like carrying or lifting heavy loads, digging or construction work for at least 10 minutes continuously? (Vigorous work activity)  2). In a typical week, on how many days do you do vigorous-intensity activities as part of your work? (Days vigorous work)  3). How much time do you spend doing vigorous-intensity activities at work on a typical day? (Minutes vigorous-intensity work) | 8.0 | PAQ605  PAQ610  PAD615 |
| 2 | Moderate work activity | 1). Moderate work activity;  2). Number of days moderate work;  3). Minutes moderate-intensity work. | 4.0 | PAQ620  PAQ625  PAD630 |
| 3 | Transportation-related activity | 1). The next questions exclude the physical activity of work that you have already mentioned. Now I would like to ask you about the usual way you travel to and from places. For example to work, for shopping, to school. Do you walk or use a bicycle for at least 10 minutes continuously to get to and from places?  2). Number of days walk or bicycle;  3). Minutes walk/bicycle for transportation. | 4.0 | PAQ635  PAQ640  PAD645 |
| 4 | Vigorous leisure-time activity | 1). The next questions exclude the work and transportation activities that you have already mentioned. Now I would like to ask you about sports, fitness and recreational activities. Do you do any vigorous-intensity sports, fitness, or recreational activities that cause large increases in breathing or heart rate like running or basketball for at least 10 minutes continuously?  2). Number of days vigorous recreational activities;  3). Minutes vigorous recreational activities. | 8.0 | PAQ650  PAQ655  PAD660 |
| 5 | Moderate leisure-time activity | 1). Moderate recreational activities;  2). Number of days moderate recreational activities;  3). Minutes moderate recreational activities. | 4.0 | PAQ665  PAQ670  PAD675 |

**Table S2. Associations of SUA levels with absolute muscle mass and height-adjusted muscle mass in the NHANES 2011–2018**

|  | **SUA levels, mg/dL** | | | | |  |  |
| --- | --- | --- | --- | --- | --- | --- | --- |
|  | **Q1** | **Q2** | **Q3** | **Q4** | **Q5** | **Per 1 mg/dL increment** | ***P* for trend** |
|  | **< 4.1** | **4.1–4.8** | **4.9–5.5** | **5.6–6.4** | **> 6.4** |  |  |
| **Low absolute muscle mass** | | | | | | | |
| Model 1 | 1 (Ref.) | 1.258 (0.917, 1.725) | 0.972 (0.701, 1.349) | 1.058 (0.734, 1.526) | 1.414 (0.843, 2.372) | 1.054 (0.953, 1.166) | 0.494 |
| Model 2 | 1 (Ref.) | 1.389 (0.985, 1.960) | 1.133 (0.795, 1.614) | 1.439 (0.980, 2.114) | 2.255 (1.333, 3.816) ** | 1.183 (1.061, 1.319) ** | 0.015 |
| **Low height-adjusted muscle mass** | | | | | | | |
| Model 1 | 1 (Ref.) | 1.330 (1.002, 1.765) * | 1.043 (0.721, 1.507) | 1.284 (0.915, 1.803) | 1.256 (0.861, 1.832) | 1.072 (0.981, 1.172) | 0.223 |
| Model 2 | 1 (Ref.) | 1.581 (1.154, 2.166) ** | 1.360 (0.923, 2.003) | 2.211 (1.454, 3.362) *** | 2.794 (1.842, 4.237) *** | 1.320 (1.196, 1.457) *** | <.001 |

Values are weighted OR (95% CI). Ref, reference. * < 0.05, ** < 0.01, *** < 0.001.

Low absolute muscle mass was defined as ALM < 19.75 kg for men and < 15.02 kg for women based on the Foundation for the National Institutes of Health (FNIH).

Low height-adjusted muscle mass was defined as ALM/height‍^2^ < 7.26 kg/m^2^ for men and < 5.45 kg/m‍^2^ for women based on the European Working Group on Sarcopenia in Older People (EWGSOP), and previous study utilizing NHANES (Karanth, Shama D et al., 2021).

All statistical analyses accounted for sample weights, strata, and primary sampling units to reflect the nationally representative estimates.

Model 1 was adjusted for age (continuous), sex, race or ethnicity, education level, family income level, smoking status, alcohol intake, physical activity, total energy intake (in quartiles), healthy eating index (in quartiles), BMI (< 25, 25–29.9, or ≥ 30 kg/m^2^) hypertension, dyslipidemia, diabetes, cancer, albuminuria, and eGFR.

Model 2 was adjusted for age (continuous), sex, race or ethnicity, education level, family income level, smoking status, alcohol intake, physical activity, total energy intake (in quartiles), healthy eating index (in quartiles), BMI (continuous), hypertension, dyslipidemia, diabetes, cancer, albuminuria, and eGFR.

**Table S3. Subgroup analysis of the association between SUA levels and relative muscle loss in the NHANES 2011–2018**

|  |  |  | **Serum uric acid levels** |  |  |  |
| --- | --- | --- | --- | --- | --- | --- |
| **Subgroups** | **Quintile 1** | **Quintile 2** | **Quintile 3** | **Quintile 4** | **Quintile 5** | **P for interaction** |
| **Age, years** |  |  |  |  |  |  |
| 18–39 | 1 (Ref.) | 0.996 (0.555, 1.786) | 0.949 (0.519, 1.734) | 1.417 (0.732, 2.743) | 1.993 (1.109, 3.582) | 0.207 |
| 40–60 | 1 (Ref.) | 0.936 (0.525, 1.671) | 1.106 (0.669, 1.829) | 0.904 (0.544, 1.502) | 1.428 (0.873, 2.337) |  |
| **Sex groups** |  |  |  |  |  |  |
| Men | 1 (Ref.) | 1.493 (0.850, 2.623) | 1.479 (0.745, 2.936) | 1.646 (0.908, 2.984) | 2.247 (1.294, 3.903) | 0.478 |
| Women | 1 (Ref.) | 1.110 (0.594, 2.074) | 1.003 (0.489, 2.057) | 0.823 (0.439, 1.543) | 1.396 (0.834, 2.338) |  |
| **Race/ethnicity** |  |  |  |  |  |  |
| Non-Hispanic white | 1 (Ref.) | 1.275 (0.592, 2.749) | 0.860 (0.396, 1.867) | 1.358 (0.709, 2.599) | 1.956 (0.997, 3.838) | 0.138 |
| Non white | 1 (Ref.) | 0.752 (0.498, 1.135) | 1.175 (0.814, 1.697) | 0.857 (0.583, 1.261) | 1.183 (0.852, 1.643) |  |
| **Educational level** |  |  |  |  |  |  |
| High school or less | 1 (Ref.) | 0.883 (0.532, 1.464) | 1.084 (0.690, 1.703) | 1.031 (0.669, 1.591) | 1.479 (0.958, 2.283) | 0.937 |
| College or above | 1 (Ref.) | 1.131 (0.611, 2.093) | 1.062 (0.574, 1.965) | 1.317 (0.767, 2.262) | 2.185 (1.210, 3.947) |  |
| **Income groups** |  |  |  |  |  |  |
| < 1.31 | 1 (Ref.) | 1.263 (0.694, 2.297) | 1.306 (0.800, 2.134) | 1.299 (0.723, 2.335) | 2.238 (1.328, 3.772) | 0.463 |
| 1.31–3.50 | 1 (Ref.) | 1.131 (0.592, 2.160) | 1.306 (0.756, 2.255) | 1.025 (0.568, 1.852) | 1.957 (1.094, 3.498) |  |
| > 3.50 | 1 (Ref.) | 0.876 (0.362, 2.120) | 0.636 (0.241, 1.677) | 0.992 (0.408, 2.409) | 1.181 (0.459, 3.039) |  |
| **Smoking groups** |  |  |  |  |  |  |
| Non-smoker | 1 (Ref.) | 0.831 (0.523, 1.321) | 0.800 (0.485, 1.321) | 0.824 (0.478, 1.421) | 1.131 (0.635, 2.014) | 0.231 |
| Smoker | 1 (Ref.) | 1.431 (0.717, 2.859) | 1.977 (0.987, 3.959) | 2.005 (1.028, 3.910) | 3.398 (1.664, 6.938) |  |
| **Alcohol intake** |  |  |  |  |  |  |
| None | 1 (Ref.) | 1.067 (0.667, 1.707) | 1.067 (0.701, 1.626) | 1.103 (0.702, 1.731) | 1.632 (1.081, 2.464) | 0.774 |
| Drinking | 1 (Ref.) | 1.714 (0.614, 4.784) | 1.886 (0.709, 5.018) | 1.712 (0.545, 5.372) | 3.449 (1.187,10.027) |  |
| **Physical activity groups** |  |  |  |  |  |  |
| Insufficient | 1 (Ref.) | 1.565 (0.875, 2.799) | 1.025 (0.530, 1.983) | 1.096 (0.511, 2.352) | 1.277 (0.647, 2.518) | 0.017* |
| Sufficient | 1 (Ref.) | 0.394 (0.084, 1.838) | 0.622 (0.187, 2.072) | 0.219 (0.045, 1.064) | 0.428 (0.120, 1.528) |  |
| Exceeding | 1 (Ref.) | 0.789 (0.446, 1.396) | 1.347 (0.687, 2.641) | 1.578 (1.044, 2.385) | 2.995 (1.666, 5.382) |  |
| **BMI, kg/m^2^** |  |  |  |  |  |  |
| < 25 | 1 (Ref.) | 2.453 (0.717, 8.390) | 2.405 (0.812, 7.121) | 5.148 (1.281,20.696) | 10.129 (2.832, 36.228) | 0.006** |
| 25–29.9 | 1 (Ref.) | 0.960 (0.445, 2.071) | 1.823 (0.849, 3.912) | 1.162 (0.471, 2.865) | 1.489 (0.596, 3.720) |  |
| ≥ 30 | 1 (Ref.) | 0.828 (0.523, 1.311) | 0.763 (0.518, 1.125) | 0.833 (0.522, 1.331) | 1.607 (1.087, 2.377) |  |
| **Hypertension** |  |  |  |  |  |  |
| No | 1 (Ref.) | 0.984 (0.595, 1.625) | 1.220 (0.806, 1.848) | 1.212 (0.737, 1.992) | 1.777 (1.118, 2.824) | 0.944 |
| Yes | 1 (Ref.) | 0.937 (0.527, 1.667) | 0.964 (0.500, 1.857) | 0.913 (0.488, 1.708) | 1.583 (0.892, 2.807) |  |
| **Dyslipidemia** |  |  |  |  |  |  |
| No | 1 (Ref.) | 1.358 (0.860, 2.144) | 1.021 (0.616, 1.695) | 1.148 (0.656, 2.011) | 1.690 (1.000, 2.855) | 0.728 |
| Yes | 1 (Ref.) | 1.035 (0.566, 1.895) | 1.208 (0.635, 2.300) | 1.067 (0.541, 2.105) | 1.864 (1.057, 3.288) |  |
| **Diabetes** |  |  |  |  |  |  |
| No | 1 (Ref.) | 0.928 (0.589, 1.464) | 1.019 (0.638, 1.628) | 1.104 (0.727, 1.676) | 1.626 (1.054, 2.508) | 0.374 |
| Yes | 1 (Ref.) | 1.562 (0.717, 3.404) | 1.003 (0.474, 2.124) | 2.024 (0.923, 4.438) | 2.662 (1.169, 6.064) |  |
| **Cancer** |  |  |  |  |  |  |
| No | 1 (Ref.) | 0.960 (0.572, 1.613) | 1.105 (0.705, 1.731) | 1.214 (0.839, 1.757) | 1.772 (1.209, 2.599) | 0.428 |
| Yes | 1 (Ref.) | 1.809 (0.335, 9.765) | 0.696 (0.089, 5.441) | 0.596 (0.053, 6.715) | 4.552 (0.614, 33.721) |  |
| **eGFR, ml/min per 1.73 m^2^** |  |  |  |  |  |  |
| < 90 | 1 (Ref.) | 0.741 (0.319, 1.720) | 1.139 (0.531, 2.444) | 0.864 (0.428, 1.747) | 1.079 (0.535, 2.178) | 0.651 |
| ≥ 90 | 1 (Ref.) | 0.979 (0.613, 1.563) | 0.909 (0.554, 1.493) | 0.854 (0.492, 1.484) | 1.180 (0.682, 2.044) |  |

Values are weighted odds ratios (95% confidence intervals). All statistical analyses accounted for sample weights, strata, and primary sampling units to reflect the nationally representative estimates. Models were adjusted for age (continuous), sex, race or ethnicity, education level, family income level, smoking status, alcohol intake, physical activity, total energy intake (in quartiles), HEI-2015 (in quartiles), BMI (< 25, 25–29.9, or ≥ 30 kg/m^2^), hypertension, dyslipidemia, diabetes, cancer, albuminuria, and eGFR. The subgroup variables themselves were not included as covariables in their respective models. * < 0.05, ** < 0.01.


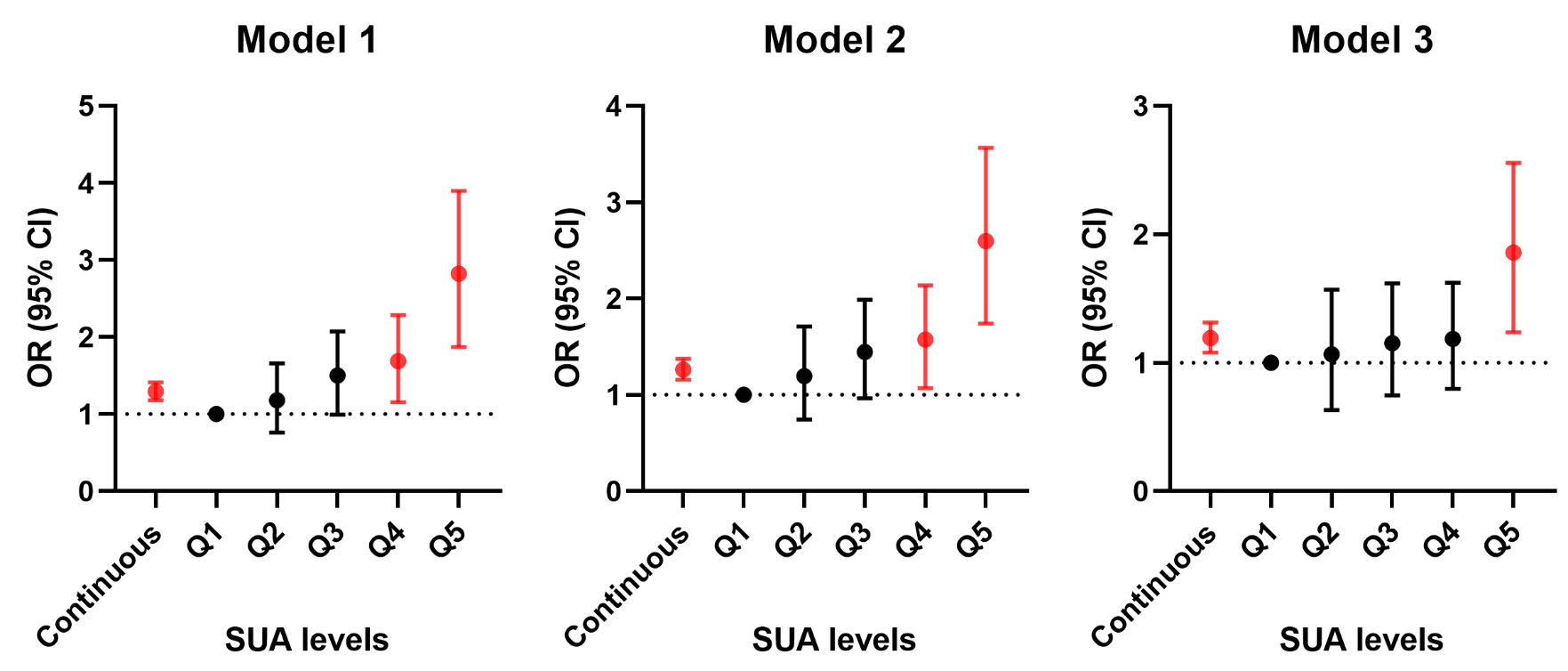


**Figure S1. Forest plot of association between SUA levels and relative muscle loss in the NHANES 2011–2018.** Quintile 1 were used as a reference. Model 1 was adjusted for age (continuous), sex, and race or ethnicity. Model 2 was further adjusted for education level, family income level, smoking status, alcohol intake, physical activity, total energy intake (in quartiles), HEI-2015 (in quartiles). Model 3 was adjusted for model 2 plus BMI (< 25, 25–29.9, or ≥ 30 kg/m^2^), hypertension, dyslipidemia, diabetes, cancer, albuminuria, and eGFR.

**
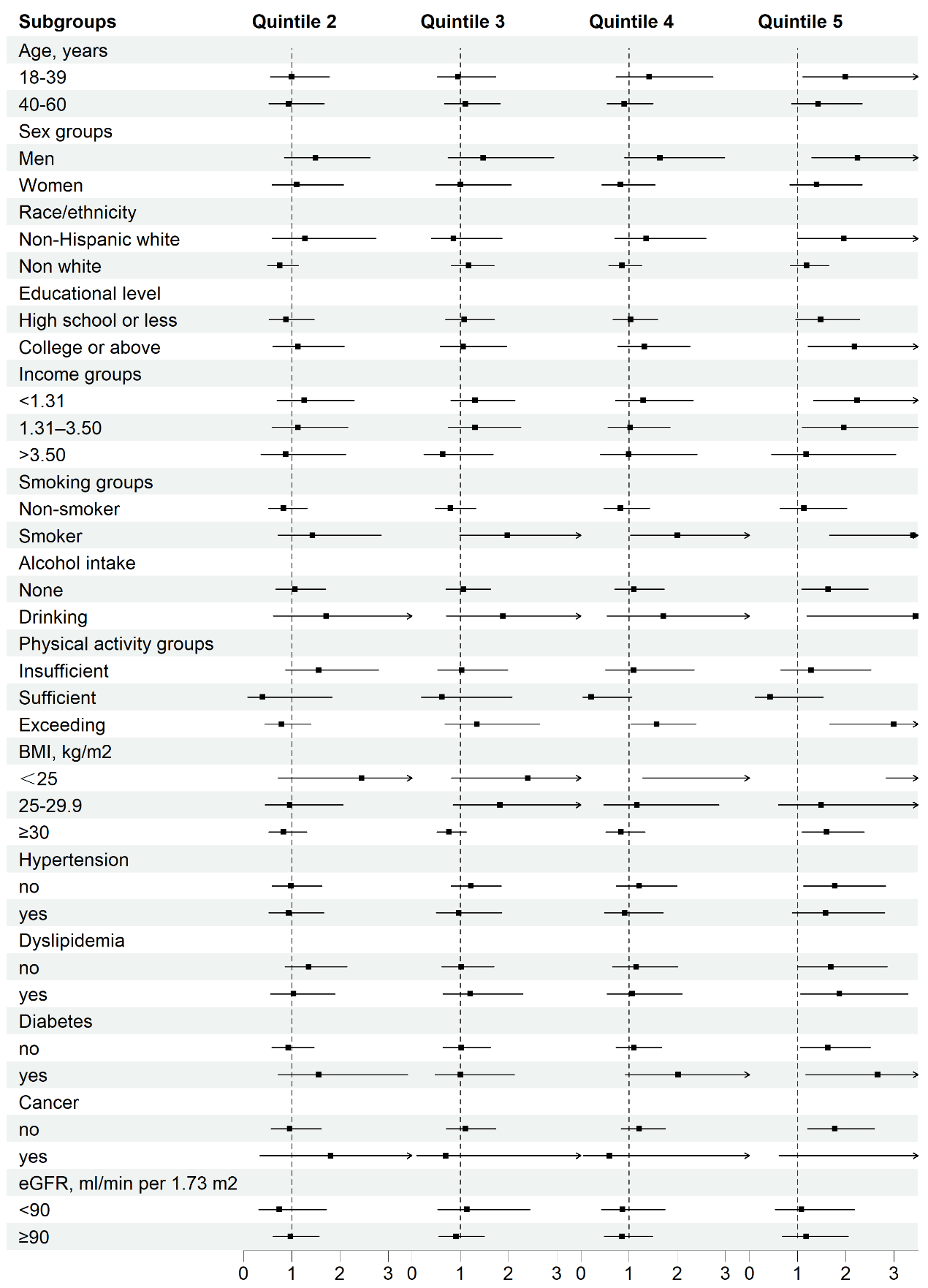
**

**Figure S2. Forest plot of subgroup analysis results on the association between SUA levels and relative muscle loss in the NHANES 2011–2018.** Quintile 1 were used as a reference.
